# RhoA: a dubious molecule in cardiac pathophysiology

**DOI:** 10.1186/s12929-021-00730-w

**Published:** 2021-04-28

**Authors:** Lucia Sophie Kilian, Jakob Voran, Derk Frank, Ashraf Yusuf Rangrez

**Affiliations:** 1 Department of Internal Medicine III (Cardiology, Angiology, Intensive Care), University Medical Center Kiel, Rosalind-Franklin Str. 12, 24105 Kiel, Germany; 2 DZHK (German Centre for Cardiovascular Research), partner site Hamburg/Kiel/Lübeck, 24105 Kiel, Germany; 3 Department of Cardiology, Angiology and Pneumology, University Hospital Heidelberg, Im Neuenheimer Feld 410, 69120 Heidelberg, Germany

**Keywords:** Rho GTPase, Actin dynamics, Signal transduction, Cell proliferation, Cardiac pathophysiology

## Abstract

The Ras homolog gene family member A (RhoA) is the founding member of Rho GTPase superfamily originally studied in cancer cells where it was found to stimulate cell cycle progression and migration. RhoA acts as a master switch control of actin dynamics essential for maintaining cytoarchitecture of a cell. In the last two decades, however, RhoA has been coined and increasingly investigated as an essential molecule involved in signal transduction and regulation of gene transcription thereby affecting physiological functions such as cell division, survival, proliferation and migration. RhoA has been shown to play an important role in cardiac remodeling and cardiomyopathies; underlying mechanisms are however still poorly understood since the results derived from in vitro and in vivo experiments are still inconclusive. Interestingly its role in the development of cardiomyopathies or heart failure remains largely unclear due to anomalies in the current data available that indicate both cardioprotective and deleterious effects. In this review, we aimed to outline the molecular mechanisms of RhoA activation, to give an overview of its regulators, and the probable mechanisms of signal transduction leading to RhoA activation and induction of downstream effector pathways and corresponding cellular responses in cardiac (patho)physiology. Furthermore, we discuss the existing studies assessing the presented results and shedding light on the often-ambiguous data. Overall, we provide an update of the molecular, physiological and pathological functions of RhoA in the heart and its potential in cardiac therapeutics.

## Background

RhoA has been originally studied in cancer cells and is well-studied with regard to its role in stimulating cell cycle progression and maintenance of actin-dynamics. However, in recent years its functions in other pathophysiological processes such as cell division, survival, proliferation and migration, signal transduction and gene transcription regulation have been emerged. In addition, in the heart, RhoA has been shown to play an important role in cardiac remodeling and cardiomyopathies. The mechanisms of the RhoA-signaling network in the heart however, are still poorly understood, because in vitro and in vivo experiments often show enigmatic results. The little existing data on RhoA function in the development and progression of cardiomyopathies show cardioprotective as well as deleterious effects of RhoA.

We thus aimed here to outline the molecular mechanisms of RhoA activation, to give an overview of the (newly) identified regulators and modulators of its activation in the heart and the probable mechanisms of signal transduction leading to RhoA activation and induction of downstream effector pathways and corresponding cellular responses in heart physiology and pathology. Furthermore, we discuss the existing studies with in vitro and in vivo experiments, assessing the presented results and shedding light on the often ambiguous data. Overall, we provide an update of the molecular, physiological and pathological functions of RhoA in the heart and its potential in cardiac therapeutics. To achieve this, a search of pertinent literature was done using online tools (PubMed, ScienceDirect and Web of Science) to screen multiple databases and by a specific search in top journals, with the following main keywords: RhoA, Rho GTPase, Rho/Rac/Cdc42, ROCK; alone and/or in combination with one or more other of these keyword(s): small GTPase, heart, cardiomyopathy, hypertrophy, GEF, GAP, GDI, GDF, GPCR-activation, PE, AngII, ET, TAC, animal-model, signal transduction, fetal genes, fibrosis, apoptosis, contractility.

## RhoA within the superfamily of small G proteins

**Fig. 1:**
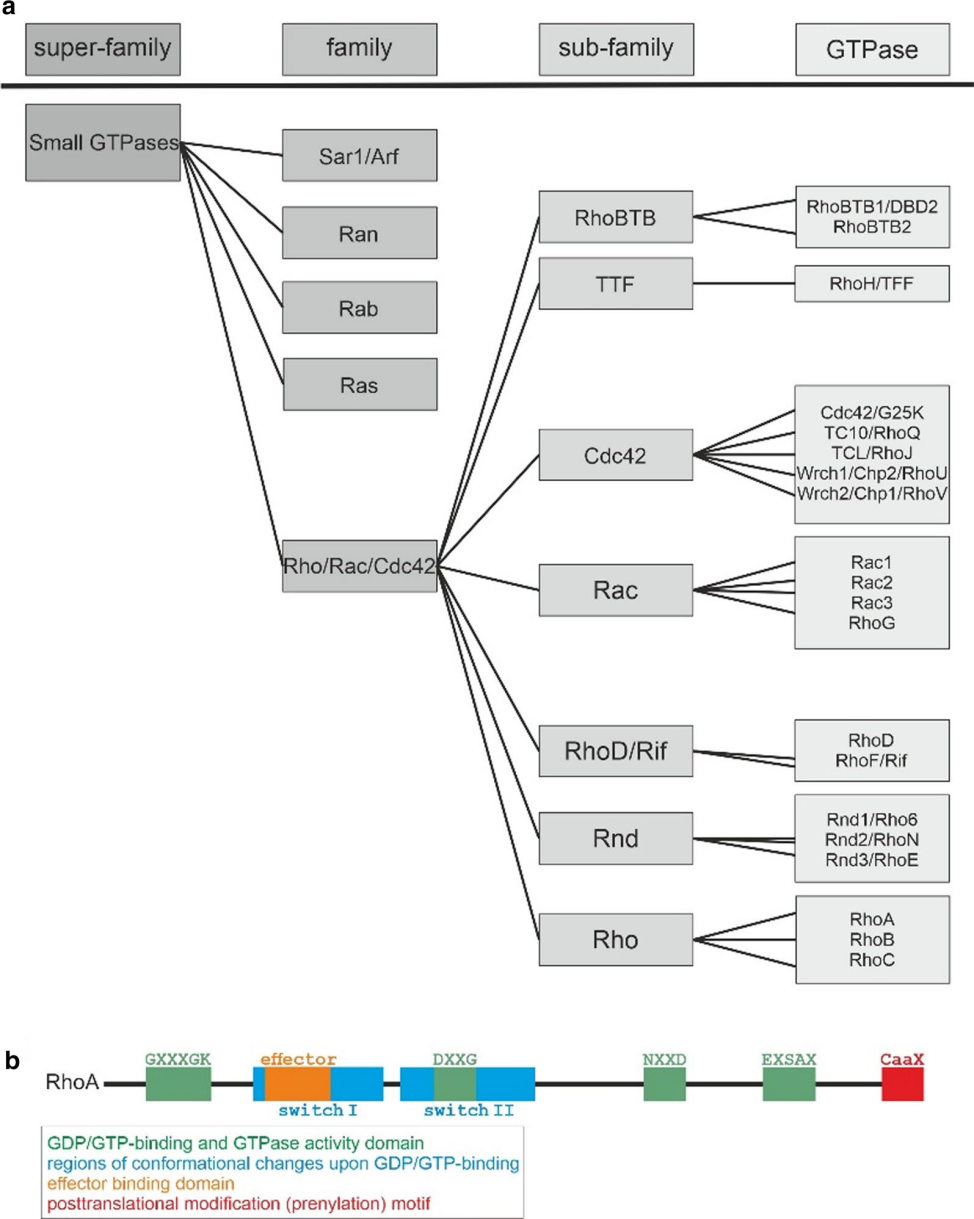
The classification of small G proteins and structural domains of the RhoA protein

Small G proteins (SmGs) are known for their essential role as molecular switches in almost all cellular processes, as for example: cytoskeletal structure, growth, mobility, proliferation, differentiation, and programmed cell death [20, 72]. They are also called “small G-binding proteins” or “small GTPases”, but although these terms seem more specific, “SmGs” is more exact and is used herein, because the GTPase activity is not necessary for their function [20, 162]. The family of SmGs includes a multitude of monomeric proteins with a molecular mass of 20–40 kDa. With more than 150 members found in humans so far it is one of the biggest protein families (Reviews: [20, 22, 96, 162]). Based on structural (amino acid sequences) and functional similarities, the members of this superfamily are generally grouped into these 5 families: (1) Ras-, (2) Rho/Rac/Cdc42- (also termed only “Rho-family”), (3) Arf/Sar1-, (4) Rab-, and (5) Ran-family [20, 22, 96, 162] (Fig. 1).

RhoA (“Ras homology family member A” or “transforming protein RhoA”) was found by cDNA screening as a “Ras homologue” in 1985 and was the founding member of the Rho-family of SmGs [103]. In the following years Rac, Cdc42, and with complete genome sequencing available, additional members were identified [34, 112]. As of today, the mammalian Rho/Rac/Cdc42-family includes 20 gene products which are found in a broad variety of species and have been studied in many model organisms from amoeba (*Dictyostelium d.)* and yeast (*Saccharomyces c.)* to zebrafish (*Danio r.*) and rat (*Rattus n.*), as well as humans (Reviews: [9, 15, 72, 130, 162]). These studies illustrated their role in the regulation of cytoskeletal organization as well as gene expression in different cell types in physiological and pathological context [162]. Besides the three eponymous subfamilies (1) Rho, (2) Rac, and (3) Cdc42, the classification of subfamilies within this group is not consistent in literature, where, up to 8 subfamilies are listed [9, 15, 162] (Fig. 1).

In humans, till date, there are ~ 150 SmGs known which are grouped into 5 families. Besides sheer numbers, the structural conservation of SmG proteins between different species indicates their functional importance. The high level of evolutionary conservation is represented in the amino acid sequence of SmGs. The Rho/Rac/cdc42-family contains 20 members, whereas, the subfamily of Rho-GTPases includes RhoA, RhoB, and RhoC. Like other SmGs, RhoA has consensus sequences for two functional domains: (1) interacting/binding with GDP/GTP and hydrolysis of GTP to GDP + P and (2) interaction with downstream effectors [16, 161] (Review: [162]) (Fig. 1). Furthermore, RhoA in mammals share a common region for posttranslational modifications (PTMs) at their carboxy-terminus (COOH) [23, 49, 52, 104, 135, 161, 181] (reviewed in: [162]) (Fig. 1).

## Posttranslational modifications

**Fig. 2:**
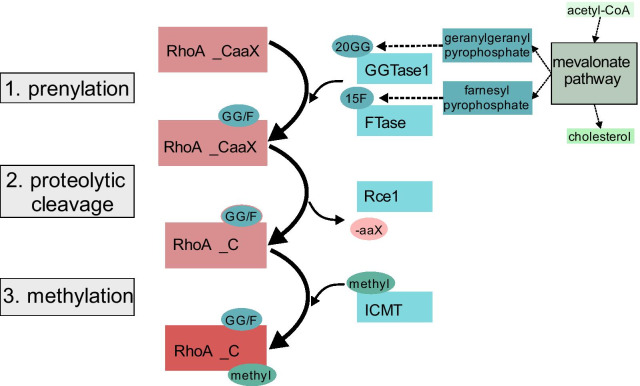
Posttranslational modifications of RhoA. Substrate is GGPP or FPP from the cholesterol generating mevalonate pathway. The first step is the prenylation by an isoprenyl-transferase (GGTase1) or the farnesyl-transferase (FTase), i.e. the adding of a 20GG- or 15F-moiety to the cysteine of the CaaX motif at the C-terminus of the RhoA-polypeptide. The second step is the proteolytic cleavage of the aaX-moieties of that motif by the protease Rce1. The last step is the methylation of the remaining cysteine by the ICMT

**Fig. 3:**
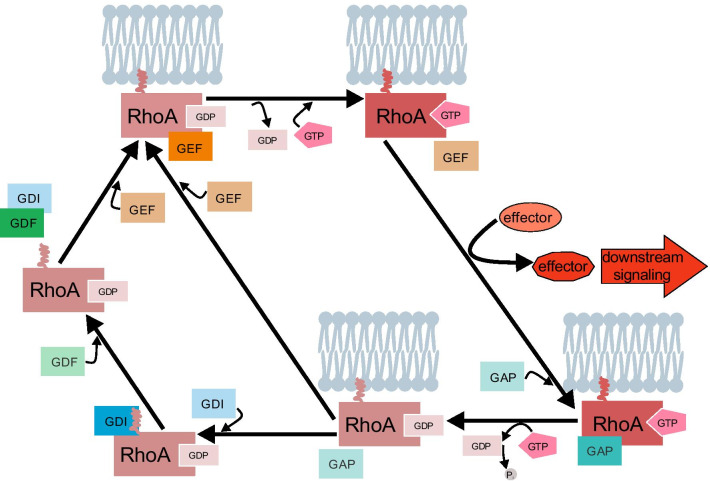
The GDP/GTP-cycle of RhoA and its regulators. RhoA cycles between a GDP-bound “inactive” and a GTP-bound “active” state. When the RhoA is anchored to the plasma membrane it is accessible for the binding of a GEF which promotes the exchange of GDP for GTP. The GTP-bound active RhoA then interacts directly with effectors, activating them and inducing downstream signaling pathways. GAPs in turn catalyze the hydrolysis of GTP to GDP (+ P_i_), bringing RhoA back into its inactive form. GDIs dislocate the RhoA-GDP from the plasma membrane, keeping it in the inactive state. GDFs on the other hand induce the dissociation of the GDIs from RhoA, allowing it’s translocation to the membrane and the start of a new GDP/GTP-cycle

As depicted in Fig. 2, PTMs, which allow the anchoring of RhoA to the cell membrane, are necessary for its activation. The region for PTMs of RhoA in mammals is a “CaaX-box-motif” at the C-terminal, a common motif for prenylation (Figs. 1 and 2) [135]. The prenylation of RhoA is essential for its function, by allowing its anchoring to the cell membrane and the binding to regulatory molecules [23, 52, 66, 104, 137, 181] (Review: [162]). The PTM of RhoA includes 3 consecutive steps: 1. prenylation, 2. proteolytic cleavage and 3. methylation (Fig. 3) [16, 52, 104, 161, 181] (Review: [162]). Upstream of these prenylation steps is the formation of geranylgeranyl pyrophosphate (GGPP) and farnesyl pyrophosphate (FPP) by synthases in the cholesterol biosynthesis mevalonate pathway [53]. The first step of the prenylation of RhoA is the linking of a 20-carbon geranylgeranyl- (GG20) or a 15-carbon farnesyl-moiety (F15) to the cysteine residue of the CaaX-motif by an isoprenyl-transferase, i.e. a geranylgeranyl-transferase (e.g. GGTase1) or a farnesyl-transferase (FTase) respectively [5, 88]. The second step is the proteolytic cleavage of the aaX-fragment by the protease Rce1 [8]. Finally, a methyl-group is added to the remaining cysteine residue via the carboxyl-methyltransferase (ICMT), forming the isoprenyl-cysteine ending [5, 8] (Fig. 2).

## Activation and inactivation of RhoA

All SmGs function as molecular switches, cycling between a GDP-bound “inactive” and GTP-bound “active” state. In the active state, SmGs can activate effectors and induce downstream signaling pathways [72]. The effectivity of this cycle and thus the activation of RhoA depend on the exchange of GDP for GTP, i.e. the formation of the GTP-bound “active” state, the hydrolysis of GTP to GDP and other positive/negative regulators which affect the localization of RhoA.

### The GDP/GTP-cycle of RhoA

RhoA cycles between two states: the GDP-bound “inactive” and the GTP-bound “active” form (Fig. 3). To start the cycle, GDP needs to dissociate from the GDP-bound RhoA anchored to the cell membrane. Once GDP is dissociated, it is replaced by GTP. The binding of GTP to the switch II region leads to conformational changes followed by changes in the switch I region (= effector loop). This enables the direct interaction of the now “active” RhoA with effector molecules and their activation [14]. The effectors are (per definition) molecules which interact (only) with the active GTP-bound state of the GTPase and get activated by it. The bound GTP is then converted to GDP + P_i_, bringing RhoA back into its “inactive” form. This conversion also leads to the release of the now activated effector protein(s) and the cycle can start again (Fig. 3).

As the affinity of RhoA for GDP is very high, the dissociation of GDP from RhoA and subsequently its replacement by GTP is slow and one of the limiting steps of RhoA-induced activation of effectors and downstream signaling pathways. Therefore, this step is usually accelerated by the mediators termed “guanine exchange factor/protein” or “guanine nucleotide releasing factor” (GEF = GEP) [14] (Fig. 3 and Table 1). The GEF interacts directly with the inactive GTPase and destabilizes its binding with GDP, leading to the dissociation of GDP [14]. The remaining GTPase + GEF complex then takes up the GTP since the GTP-concentration in the cytoplasm is generally higher than the GDP-concentration. The conformational changes induced by the GTP-binding lead to the effector activation and the release of the GEF. The second limiting step is the hydrolysis of GTP (back) to GDP + P_i_. Although RhoA as a GTPase has an intrinsic hydrolytic activity and can perform the reaction on its own, it is mostly facilitated by a so called “GTPase activating/-accelerating protein” (GAP), which catalyzes the hydrolysis [14]. Thus, the release of the activated effector is induced and a new round of the activation-cycle can start (Fig. 3). Besides GEFs and GAPs, the SmGs of the Rho/Rac/Cdc42- (and Rab-) family are regulated by two more groups of regulatory proteins: “guanine nucleotide dissociation inhibitors” (GDIs) and “GDI dissociation/displacement factors/proteins/stimulators” (GDFs = GDPs = GDSs) (Table 1). GDIs keep RhoA in its GDP-bound inactive state by disconnecting it from the plasma membrane. They bind to the prenylated C-terminus, disrupting the anchoring to the membrane and detaining it in the cytoplasm, inaccessible for the GEFs [48]. In contrast, GDFs catalyze the dissociation of the GDIs from RhoA. RhoA can then anchor to the membrane and is accessible for GEFs and subsequently the GAP-mediated exchange from GDP to GTP starts again [35, 63] (Fig. 3).

**Table 1:** GEFs, GAPs, GDIs, and GDFs of RhoA

Group	Regulator name in human (mouse)	Interaction with SMGS	Domains	Receptor activation	References
GEFs	Dbl	Rho/Rac/Cdc42	DH/PH	(Gαq/11)	[56, 136, 174]
	Dbs	Rho, Cdc42	DH/PH	(Gαq/11)	[136, 171]
	LARG-RhoGEF (ARHGEF12)	Rho	DH/PH, PDZ	Gα12/13	[3, 159]
	Lbc (AKAP/ARHGEF13)	Rho	DH/PH	Gα12/13	[3, 183]
	Lfc (ARHGEF2/GEF-H1)	Rho	DH/PH	Gα12/13	[50, 107]
	p63RhoGEF, *GEFT	Rho	DH/PH	Gαq/11	[100, 136]
	p115RhoGEF (ARHGEF1/Lsc)	Rho	DH/PH, RH	Gα12/13	[50, 57, 85]
	PDZ-RhoGEF (ARHGEF11)	Rho	DH/PH, RH, PDZ	Gα12/13	[3, 46]
	Vav1	Rho/Rac/Cdc42	DH/PH, SH2	Src	[28]
	Vav2	Rho	DH/PH, SH2	Src	[145]
Group	Regulator (name)	Interaction with SmGs	Model		Reference
GAPs	Graf	Rho, Cdc42	Chicken embryo (CE) cells		[62]
	Dlc1	Rho/Rac/Cdc42	Tumor cells (liver, HeLa)		[177]
	Myr5	Rho, Cdc42	Isolated GTPases		[129]
	RA-RhoGAP	Rho	Smooth muscle		[184]
	ARAP3	Rho, Arf	Smooth muscle		[184]
	p50RhoGAP	Rho, Rac, Cdc42	Kidney cells (HEK), fibroblasts (COS-7)		[11, 87]
	p190RhoGAP	Rho Rac, Cdc42	Tumor cells (HeLa), fibroblasts (COS-7), neurons		[7, 59, 132]
Group	Regulator (name)	Interaction with SmGs	Model		Reference
GDIs	RhoGDI1 (RhoGDIα)	Rho/Rac/Cdc42	Neurons		[166]
	*RhoGDI2 (RhoGDIβ/D4/ Ly-GDI)	Rho/Rac/Cdc42	Lymphocytes		[92, 144]
	*RhoGDI3 (RhoGDIγ)	RhoB and RhoG (not RhoA and RhoC)	Tumor cells (HeLa)		[180]
Group	Regulator (name)	Interaction with GDIs	Model		Reference
GDFs	ERM	RhoGDIα	Fibroblasts (COS-7)		[160]
	p75NTR	RhoGDI	Neuroblasts (PC12)		[175]
	IΚΚγ/NEMO	RhoGDI	HEK cells (293 T)		[80]

Listed are the GEFs, GAPs, GDIs and GDFs which have been shown to interact with RhoA. For the GEFs the SmG-(sub-) family with which members they interact besides RhoA is listed, as well as the conserved domains which are responsible for the interaction with the SmG and the mechanism of activation. For the GAPs, GDIs and GDFs also the interaction partners and the cell model in which the activity on RhoA has been shown are listed. (* = isoform)

### The network of regulators of RhoA activation

**Fig. 4:**
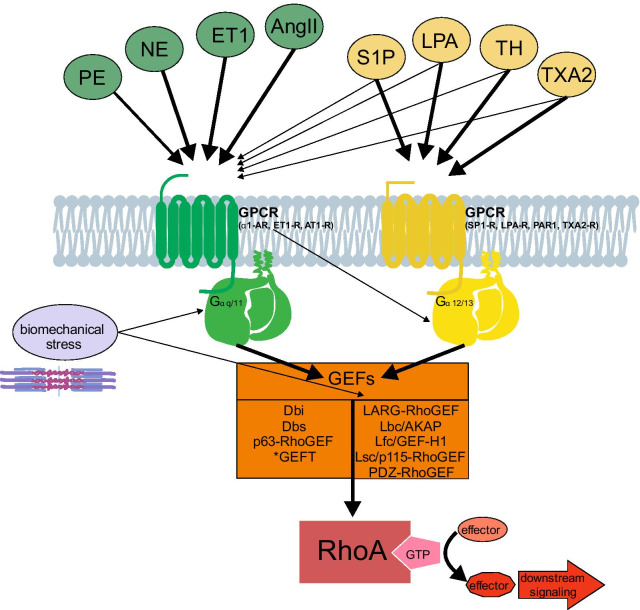
Activation of RhoA by ligand-induced GPCR activation and Gα-GEF interaction. A link between RhoA-activation and extracellular stimuli is the binding of ligands to GPCRs which activate Gαq/11 or Gα12/13 that in turn act as activators for RhoGEFs. In addition, biomechanical stress is also linked to Gαq/11, GEF and RhoA-activation

There are 10 GEFs that have been shown to interact directly with Rho-subfamily members so far (Table 1). In the last years there have been studies to analyze the mechanisms of their activation and an interaction with G proteins coupling to “G-protein coupled receptors” (GPCRs) have been shown to play an important role for the activation of most of them (Table 1). For example the RhoA-GEF p115RhoGEF was the first, for which the localization to the membrane and interaction with GPCR-coupled Gα12/13 has been confirmed to be essential for its activation [85] (Table 1 and Fig. 4). Vav1 and Vav2 on the other hand are examples for GEFs which need PTMs to get activated. Phosphorylation by the Scr tyrosine kinase allows them to bind with their SH2-domains to RhoA and activate the GTPase [81] (Table 1). Some GEFs have additional domains for protein–protein interaction such as a SH3-domain (Vav2, Dbs) and might have additional signaling functions [162].

Though GAPs mainly function as negative regulators, they can also be seen as positive mediators, because by accelerating the hydrolysis of GTP to GDP and promoting the release of the bound effector, they make a new round of GDP exchange with GTP and the binding of a new effector possible. Like GEFs, some GAPs interact with a variety of SmGs (e.g. p190RhoGAP interacting with different Rho/Rac/Cdc42-family members) [152], but in general GAPs are also specific, interacting only with members of one subfamily (e.g. Rab3-GAP for Rab3A/B/C) [47]. There are more than 85 GAPs known for the Rho/Rac/Cdc42-family, of which, 7 GAPs have been shown to interact with Rho-subfamily members (Table 1). The best studied of these GAPs are p50RhoGTPA and p190RhoGAP, which bind to a variety of Rho/Rac/Cdc42-family members in vitro, but preferably to Rho-GTPases in vivo [87, 132] (Table 1). Some GAPs seem to allow a crosstalk between otherwise distinct signaling pathways of members of different SmG-families. For example p190RhoGAP has been shown to promote the activity of the p120RasGAP [67], whereas p120RasGAP in turn has been shown to inhibit the Rho-GAP Dlc1 in tumor cells [177]. Furthermore, p190Rho-GAP has been shown to be activated and reduce RhoA-activity in a Rac1-dependent manner in tumor cells and fibroblasts [59, 116] and RA-Rho-GAP and ARAP3 are activated by binding of Rap1, probably inducing the downregulation of RhoA in smooth muscle cells [184] (Table 1).

GDIs are negative regulators of RhoA, as they detain RhoA in the cytoplasm and in the inactive state, whereas, GDFs on the other hand are positive regulators, because they dissociate GDIs from RhoA, making the GTPase accessible for GEFs again [35]. GDIs in general are less specific than GEFs and GAPs, interacting with most Rho/Rac/Cdc42- and Rab-family members [63, 93, 166, 167]. The known RhoGDIs for Rho-subfamily members are RhoGDIα (Rho-GDI1 or only RhoGDI) and its two isoforms RhoGDIβ (RhoGDI2 or D4/Ly-GDI) and RhoGDIγ. RhoGDIα and RhoGDIβ have been shown to interact with RhoA, while RhoGDIγ interacts with other Rho-subfamily members, but not RhoA [180] (Table 1). The importance of RhoGDIα in the heart has been established using transgenic mice overexpressing RhoGDIα in a cardiomyocyte-specific manner, where, the amount of RhoA linked to the plasma membrane was reduced while the amount in the cytosol was highly increased [20].

There are only 3 known GDFs of Rho-subfamily members: ERM, p75NTR and IΚΚγ. It has been shown that ERM interacts with RhoGDIα and weakens its binding to RhoA in fibroblasts [160], and p75NTR and IΚΚγ interact with RhoGDIα in neuroblasts and HEK-cells, respectively, also promoting the RhoA activation [80, 175].

The big number of GEFs and GAPs make it difficult to analyze the role of specific positive or negative effectors of RhoA in different signaling pathways. Moreover, systematic studies that discriminate the effect of different GEFs or GAPs in pathophysiological signaling of cardiomyocytes are not published yet. For GDIs, only limited data is available such as the mouse model with cardiac RhoGDIα overexpression [20]. This model has also shown that there are feedback loops that lead to upregulation of RhoA-expression, if the amount of inactive RhoA (in the cytosol) is increased [20]. The cardiac functions of GDFs in RhoA signaling are not explored yet. Overall, there is still a grey zone and many unexplored territories if and how these GEFs, GAPs, GDIs and GDFs affect cardiac function in general and RhoA signaling in particular.

## Cardiac functions of RhoA: lessons learnt from in vitro and animal models

RhoA is ubiquitously expressed in human tissue. The RhoA protein is well known for its role in stress fiber formation, first described by Ridley and Hall in 1992 in 3T3-fibroblasts [131]. RhoA also plays an essential role in embryonic development of the heart by influencing (pre-) cardiomyocyte proliferation and differentiation [73]. In recent years, its role in pathophysiology of the heart has come more into focus. Interestingly though, as more and more cardiac upstream regulators and downstream effectors of RhoA are known, many studies show contradictory associations of RhoA, due to its diverse network of signaling pathways. On one hand, RhoA is associated with induction of pathological cardiac hypertrophy and re-activation of pro-hypertrophic fetal genes [76, 139, 164]; on the other hand, data suggests a cardio-protective role for RhoA by inducing cell survival signaling [115]. Experiments with mouse models and pressure overload-induced hypertrophy indicate that RhoA is necessary to prolong the transition from adaptive to maladaptive hypertrophy with dilation or heart failure [90].

As early as in 1999, it was shown that mice overexpressing RhoA (~ 20-fold) from early development in a cardiac-specific manner develop dilated cardiomyopathy with heart failure and die within a few weeks of age [140]. Nevertheless, in a more recent study milder cardiac-specific RhoA-overexpression (~ fivefold) induced in adult mice does not lead to increased lethality nor to a pathological cardiac phenotype [173]. In experiments with knockdown of RhoA, it has been shown that RhoA is necessary for the maintenance and reorganization of cell–cell contacts in cardiomyocytes in vitro [158]. Furthermore, our group has shown that neonatal rat cardiomyocytes (NRVCMs) infected with “control-virus” (lacZ) and treated with C3-transferase, a known RhoA-specific inhibitor, exhibit reduced cell size and disrupted actin cytoskeleton structure, while SRF-activity and proliferation marker expression were not affected [127]. Lauriol et al. have shown that knockout of RhoA itself in adult mice does not lead to a pathological phenotype under physiological or acute pressure overload conditions, however, chronic pressure overload in these mice led to a severe contractile dysfunction associated with aberrant calcium signaling and decreased activity of ERK1/2 and AKT [90]. Pressure overload-induced hypertrophy by TAC in these mice led to shift from thickening of the LV-wall, and consequently smaller LV diameter (LVD) and increase in LV pressure (LVP) in the control mice, to dilation with thinning of the LV-wall and increase in LVD, LVP and LV-volume (LVV) in the knockout mice [90]. This indicates an earlier transition from adaptive to maladaptive hypertrophy. In the stage of heart failure, the dilation was even bigger and the impairment of the pumping capacity (fractional shortening (FS)) stronger than in the control mice [90]. Absence of RhoA in the heart did not affect the TAC-induced upregulation of the fetal genes *NppB* and *MYH7*, which are generally associated with hypertrophy and heart failure [90]. However, RhoA-deficiency blunted the TAC-induced upregulation of the F-/G-actin ratio as well as MERTF-A and SRF expression, which is in line with a role of RhoA-signaling in actin-polymerization. Furthermore, the RhoA-knockout diminished the TAC-induced fibrosis and apoptosis associated with the severe stage of heart failure [90]. Taken together, these results underline the importance of RhoA-signaling in actin-polymerization in healthy cardiomyocytes and under pressure-overload induced hypertrophy. Moreover, they also show a cardio-protective role of RhoA by (extending) compensatory hypertrophy and prolonging the transition to pathological hypertrophy, i.e. fibrosis and heart failure [90]. In addition, the results indicate that RhoA-independent signaling is sufficient for TAC-induced up-regulation of hypertrophic/fetal genes (*NppB* and *MYH7*).

Taken together, the few published studies show that the effects of RhoA (over-) expression are highly dose dependent and that RhoA is essential for the maintenance of cytoskeletal organization and maintenance of cardiac homeostasis under stress. The molecular signaling pathways behind the effects of RhoA in hypertrophic responses are not completely understood yet, but the activation of RhoA in different models of hypertrophy with biomechanical or a variety of hormonal stimulations is well known. GPCRs and two groups of G-proteins coupling to them and acting as GEFs for RhoA have come into focus as inducers of RhoA activation, as discussed below.

### RhoA-activation via G-protein coupled receptors in cardiac hypertrophy

Extracellular hypertrophic stimuli start intracellular signaling cascades of RhoA in cardiomyocytes by binding to GPCRs [20, 100, 108, 136]. These receptors are usually activated by binding of ligands (e.g. hormones) on the cell surface. This induces a conformational change in their intercellular domain that activates the α-subunit of the coupled G-protein, which then activates downstream effectors [3]. In humans, 4 different types of α-subunits of GPCRs are known. Of these GPCR-coupling G-proteins with the subunits Gαq/11 (Gαq and its homologue Gα11) and Gα12/13 (Gα12 and its homologue Gα13) have been shown to play a role in the heart [108]. Different G-proteins binding to certain GPCRs and activating downstream effectors provide a degree of specificity for the biological response connected with a variety of GPCR-binding agonists [20]. They are involved in a broad variety of physiological and pathological signaling pathways.

The first group of ligands whose binding to their respective GPCRs has been connected with RhoA-activation includes: phenylephrine (PE), norepinephrine (NE), endothelin 1 (ET1) and angiotensin II (AngII). The binding of these ligands to their corresponding receptors α1-adrenergic receptors (α1-AR), ET1-receptors (ET1-R) or angiotensin II-receptors (mainly AT1-R) efficiently activate Gαq/11 (Table 1 and Fig. 4) [20]. All these agonists are well established inducers of cardiomyocyte hypertrophy in vitro and in vivo. The cellular responses in NRVCMs which are triggered by this signal transduction pathway are an increase of cardiomyocyte size and protein synthesis, as well as actin polymerization and re-activation of fetal genes (such as *NppA*, *NppB* and *MYH7*) associated with pathological hypertrophy [43, 84, 176]. In vivo the responses of this (chronic) stimulation are cardiomyocyte growth as seen in an increase in heart size (and weight) and then, when the (adaptive) hypertrophy transitions into pathological hypertrophy with dilation and heart failure, increase in the left ventricle diameter, thinning of the left ventricle wall, increased fibrosis (and apoptosis) as well as reduction of ejection fraction and fractional shortening [43, 58].

Using a gain-of-function approach, Gαq has been shown to act as a pro-hypertrophic molecule, both in vitro and in vivo [1, 29]. The involvement of RhoA in this signaling became clear, when inhibition of RhoA by treatment with C3-transferase, transfection with anti-sense oligonucleotides for RhoA, or expression of a non-functional from of RhoA, attenuated the hypertrophic responses of NRVCMs after stimulation by PE-treatment or stretch [76, 139, 164]. Nevertheless, the mechanism by which GPCR and subsequent Gαq-activation lead to RhoA-activation was long not understood. Now it is clear that specific Rho-GEFs can provide this critical link. Recently, a direct interaction between Gαq/11 and the p63-RhoGEF, a GEF of RhoA, has been shown, providing the long missing link between the GPCR-activation by ligands and RhoA-activation [100, 136]. Although the interactions of Gαq/11 and RhoGEFs on molecular level are known, experiments for analyzing the exact functional impact of these interactions in the context of cardiomyocyte hypertrophy have not been done yet, likely due to the fact that, up to now, no specific inhibitors of these GEFs are available.

The second group of ligands that activate RhoA very effectively bind to GPCRs, which can couple to Gαq/11 as well, but couple with higher affinity to Gα12/13. This group of ligands includes: sphingosine-1-phosphate (S1P), lysophosphatidic acid (LPA), thrombin (TH) and thromboxane 2 (TXA2) with their respective GPCRs: SP1-receptor (S1P-R), LPA-receptor (LPA-R), protease-activated receptor (PAR, mainly PAR1) and TXA2-receptor (TXA2-R) [134] (Fig. 4).

A study also showed the activation of Gα12/13 by the α1-AR [105]. S1P and LPA can also induce cardiomyocyte hypertrophy (i.e. increase of actin organization and fetal gene expression) in vitro, but the hypertrophic response is much less robust than for PE, ET1 or AngII [61, 169]. In addition S1P is known for its cardio-protective function in ischemia/reperfusion models [163]. Involvement of Gα12/13 in pro-hypertrophic signaling has been shown by experiments where overexpression of Gα12 in NRVCMs lead to a hypertrophic response, i.e. an increase in cell size [40]. The connection between the activation of receptors coupling to Gα12/13 and RhoA activation has been shown by experiments where S1P receptor activation led to RhoA activation [70]. LPA has also been shown to activate RhoA (and its effector ROCK I) via Gα12/13 [126]. As with Gαq/11, a subset of Rho-GEFs interacts directly with the activated Gα12/13, get activated themselves and then induce the activation of RhoA (as described above: Figs. 3, and 4).

The Rho-GEFs which have been shown so far to be activated by Gα12/13 stimulation are: p115RhoGEF, LARGE-RhoGEF, PDZ-RhoGEF and AKAP-Lbc and Lfc/GEF-H1 [3, 6, 85, 107, 159] (Table 1 and Fig. 4). Appert-Collin et al. have shown that activation of AKAP-Lbc mediates hypertrophy in PE-stimulated NRVCMs via the ligand binding to α1-AR and subsequent activation of Gα12 and AKAP-Lbc [6].

There is increasing evidence that molecular signaling cascades induced by mechanical stress in cardiomyocytes also work partly via Gα-activation, although data on the involvement of Gαq/11 and Gα12/13 are contradictory as the underlying mechanisms are not clear yet. With regards to Gαq/11, the hypertrophic responses (i.e. fetal gene expression and increased heart weight) to TAC stimulation in mice were reduced by depletion/inhibition of Gαq/11 [4, 170]. Whereas, for Gα12/13, S1P-treatment or Gα12/13 overexpression in NRVCMs induced RhoA activation and hypertrophic responses [40, 61, 169], nevertheless, depletion/inhibition of S1P-R2/ S1P-R3 or Gα12/13 had no effect on TAC-induced hypertrophic responses in mice [32, 33, 117]. Seemingly contrary, the Gα12/13 inhibition did reduce stretch-induced RhoA-activation and expression of fibrosis markers in stretched NRVCMs and TAC-operated mice [117]. This fits to the in vivo experiments mentioned above, where RhoA knockdown did not affect fetal gene expression, but did reduce fibrosis [90]. Moreover, LARG-RhoGEF and GEF-H1, previously associated with Gα12/13 activation [107, 159], have been shown to get activated by mechanical force on integrins, leading to activation of RhoA [54]. Collectively, these reports suggest that Gαq/11-dependent, but Gα12/13-independent activation of RhoA might play an important role in pressure-overload induced cardiac hypertrophy. On the other hand Gα12/13-dependent RhoA-signaling seems to be involved in fibrotic responses to mechanical stress, further adding to the complexity or RhoA signaling, where more than one signaling pathway with different GPCRs/G-proteins (Gαq/11 and/or Gα12/13), and even G-protein independent pathways are activated (in parallel) by biomechanical stress (in vivo) (Fig. 4).

### Upstream modulators of RhoA in cardiac hypertrophy

**Fig. 5:**
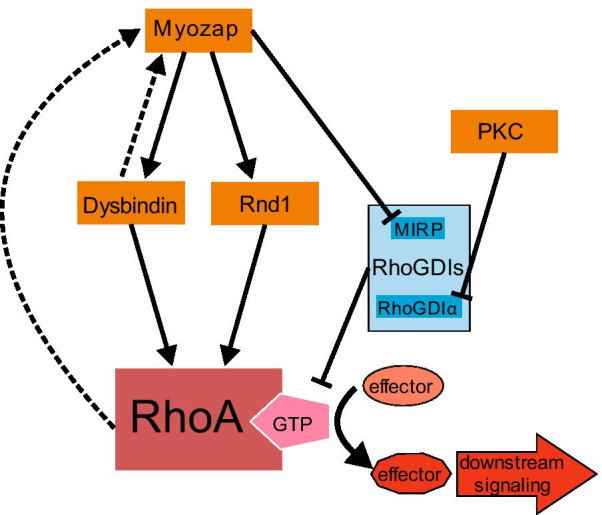
Upstream modulators of RhoA signaling in cardiomyocytes. In recent years these upstream modulators and (direct) interaction partners of RhoA in the context of cardiac hypertrophy have been found: Myozap can promote RhoA activation and downstream effector signaling via Dysbindin activation, Rnd1-activation, and blocking of the RhoA-inhibitor (GTP-dissociation inhibitor) MIRP. PKC can promote RhoA-activation via inhibition of the RhoA GTP-dissociation inhibitor RhoGDIα

Besides the regulation of the GDP/GTP-cycle by Gα-GEF-interaction, a number of other proteins have been found to directly influence RhoA-activation and hypertrophic signaling (Fig. 5). Dysbindin (dystrobrevin-binding protein) for example has recently been identified by our group as a direct interaction partner of RhoA in cardiomyocytes in vitro [127]. Overexpression of Dysbindin in NRVCMs has been shown to induce cellular hypertrophy via RhoA-dependent SRF-activation [127]. Consistently, inhibition of RhoA blocked the pro-hypertrophic effects of the Dysbindin overexpression, while knockdown of Dysbindin reduced the hypertrophic effects and the SRF-activation, induced by PE and ET1-treatment [127]. So, we identified Dysbindin as an upstream mediator of RhoA-signaling (Fig. 5). The Dysbindin-promoted RhoA-induced hypertrophic responses were mediated, at least partly, via a NFAT-independent pathway, as inhibition of NFAT did not block these responses [127]. Probable NFAT-independent signaling pathways include SRF/CArG and/or YAP/TEAD activation. Dysbindin also links RhoA to Myozap. Overexpression of Myozap, an ERM-domain containing protein, has been shown to induce hypertrophy in vitro and in vivo via SRF-activation [127, 151]. Downregulation of Dysbindin reduced this Myozap-induced SRF-activation in vitro [127] (Fig. 5). Other ERM-domain containing proteins have also been shown to regulate RhoA by functioning as a GDF, binding with this region to RhoGDIs and initiating their dissociation from RhoA, thus promoting the RhoA activation [160] (Fig. 3). A direct interaction between Myozap and RhoA has not been shown, but Myozap binds the “Myosin phosphatase-RhoA interacting protein” (MRIP, p116RIP in mice), a RhoA inhibitor, via its ERM-domain [151]. So, Myozap might function as a GDF, promoting RhoA-activation by abolishing the inhibitory action of MIRP which functions as RhoGDI. In addition Myozap might also be affected by downstream-signaling of RhoA-activation, as ERM-proteins are often activated by RhoA-dependent phosphorylation [165]. This might speak for a positive feedback loop (Fig. 5).

Another protein that has just recently been described to be connected with RhoA-activation, Dysbindin, and Myozap is Rho-family GTPase 1 (Rnd1). Rnd1 is another member of the Rho/Rac/Cdc42-family of SmGs (Fig. 1). Rnd1 has been shown by our group to be upregulated in various in vitro and in vivo models of cardiac hypertrophy (PE- and stretch-stimulated NRVCMs and TAC-operated mice) [83]. Overexpression of Rnd1 in NRVCMs led to increased levels of active RhoA, upregulation of SRF-expression and -activation, and hypertrophy. Inhibition of RhoA with C3-transferase on the other hand abolished all those hypertrophic responses [83], indicating that like Dysbindin, Rnd1 is also an upstream modulator of RhoA (Fig. 5). As knockdown of Rnd1 blunted the SRF-activation induced by Myozap-overexpression, whereas Myozap-knockdown did not reduce the Rnd1-induced SRF-signaling, Myozap in turn has been proposed to act as an upstream activator of Rnd1 [83]. Thus, we postulate that Myozap could act in three ways as RhoA-activator: firstly via activation of Dysbindin, secondly via activation of Rnd1 and thirdly by blocking of MIRP (Fig. 5).

The mediation of RhoA-activity by interacting with a Rho-GDI has also been described for the protein kinase C (PKC) (Fig. 5). Overexpression of PKCα has been shown to induce pro-hypertrophic gene expression and cardiomyocyte growth [17]. In endothelial cells, PKCα-signaling has been shown to increase thrombin-induced activation of RhoA by phosphorylation and subsequent dissociation of Rho-GDIα from RhoA [106]. This phosphorylation of RhoGDIα also occurred in stretched NRVCMs [122]. Because the induction of RhoGDIα-phosphorylation, as well as RhoA-activation by stretch was reduced by PKC-inhibition, the function of PKC as another upstream modulator of RhoA has been proposed [122]. Looking at the network of signal-transduction and the number of downstream effectors/pathways/cellular responses of RhoA activation, there are surely more upstream-modulators of RhoA, yet to be discovered.

### Downstream molecules in RhoA-induced pathological signaling

A dozen of direct RhoA effectors are known so far. As every single one of these effectors activates multiple downstream signaling molecules, the complexity of the RhoA-regulated network of signaling pathways is highly immense. The Rho-kinases (ROCK) have been intensively studied and there are a number of excellent reviews on their function in cardiac pathophysiology (Reviews: [5, 99]). In contrast, the role of other effectors in cardiomyocytes has only been studied superficially so far. In the next paragraphs, a selection of effectors and their role in cardiomyocyte hypertrophic signaling pathways is described in detail.

**Fig. 6:**
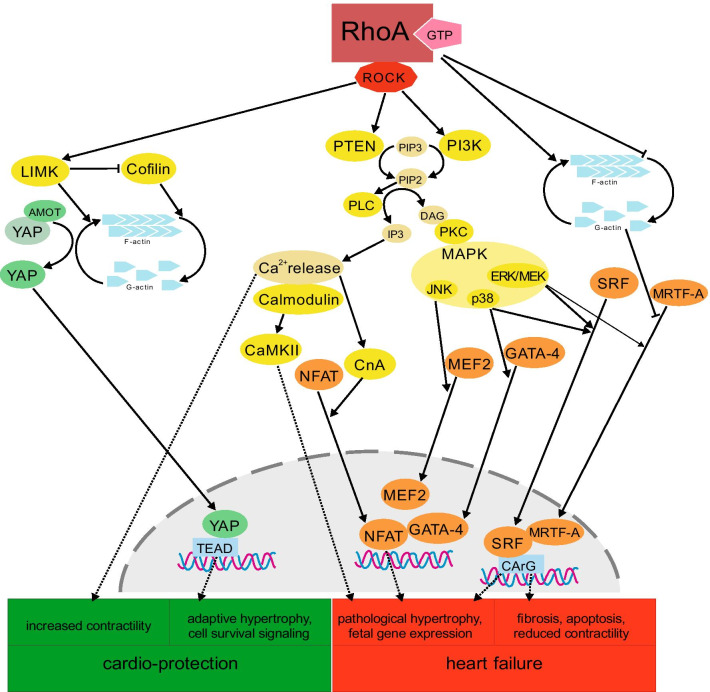
Effectors of RhoA in cardiomyocyte hypertrophic signaling. In cardiomyocytes RhoA activation leads to the activation of cardio-protective signaling, but also signaling associated with heart failure. ROCK is the best studied downstream effector of RhoA. RhoA/ROCK activates LIMK, which promotes F-actin formation and stabilization via inhibition of cofilin. Interaction of AMOT-bound YAP with F-actin leads to release and translocation of YAP into the nucleus, and subsequently to the expression of genes for cell survival and adaptive hypertrophy. In contrast, the RhoA-induced reduction of G-actin promotes the translocation of MRTF-A into the nucleus, promoting pathological signaling. RhoA/ROCK activates PTEN and PI3K which convert PIP3 to PIP2, thus promoting PLC activation. PLC in turn converts PIP2 to IP3 and DAG. IP3 can stimulate the release of Ca^2+^ into the cytoplasm, which increases cardiomyocyte contractility. Besides, the increased Ca^2+^-level activate Calmodulin/CaMKII and CnA, inducing pathological signaling. DAG activates PKC and subsequently the MAPKinases JNK, p38 and ERK/MEK, which in turn promote the translocation of MEF2, GATA-4, SRF and MRTF-A into the nucleus and subsequently the expression of genes leading to pathological hypertrophy with heart failure

The best studied downstream effectors of RhoA playing a role in cardiac hypertrophy are the two isoforms of the “Rho-associated coiled-coil containing protein kinase” ROCK-1 (ROKβ) and ROCK-2 (ROKα). However, experiments analyzing the function of ROCK give seemingly contrary results. On the one hand treatment with fasudil (HA-1077), a specific ROCK inhibitor, was shown to reduce pressure overload-induced hypertrophy and fibrosis in TAC-operated mice [125] as well as AngII-stimulated rats [60]. On the other hand, ROCK1 depletion in mice did not reduce hypertrophy after TAC- or AngII-stimulation in mice, but only fibrosis [133, 182]. The role of ROCK1 signaling was also analyzed in Gαq-overexpressing mice (a model for dilated cardiomyopathy) crossbreed with ROCK1-knockout mice. The resulting Gαq-overexpressing, but ROCK1-knockout mice showed lesser impairment of cardiac pumping capacity after TAC [154]. This suggests that ROCK acts downstream of Gαq-activation and promotes fibrosis upon hypertrophic stimulation. These contrasting results regarding RhoA/ROCK signaling in cardiac hypertrophy may be explained by differences in the states of hypertrophy, i.e. compensatory or pathological hypertrophy. Taken together the results suggest that RhoA/ROCK1 pathways play a main role in the severe state of hypertrophy, which is associated with dilation, fibrosis and heart failure. However, in in vivo models with conventional knockout or inhibition of RhoA/ROCK, the effects on cardiomyocytes cannot be specified completely, because, RhoA/ROCK affects other cell types as well [20, 108]. Especially in fibroblasts, RhoA induces proliferation and migration, which could account for example for the effects regarding fibrosis and thus the promotion of transition from adaptive to pathological hypertrophy with heart failure [20]. On molecular level a large number of signaling molecules associated with pathological hypertrophy are known to be regulated by ROCK. Experiments in mice have shown that “Calcium-Calmodulin-dependent kinase 2” (CaMKII) is necessary for pressure overload-induced hypertrophic responses [10] and that Calcineurin (Calcineurin A = CnA) depletion in mice blocks the hypertrophic responses generally induced by pressure overload or agonist stimulation (e.g. AngII infusion) [21]. The RhoA effector ROCK activates PTEN and PI3K which convert phosphatidylinositol-3,4,5-triphosphate (PIP3) to phosphatidylinositol 4,5-bisphosphate (PIP2) [90]. An increase in PIP2-level leads to activation of PLC which then hydrolyses PIP2 to diacyl glycerol (DAG) and IP3 (inositol trisphosphate). IP3 can bind Ca-channels and induce the release of Ca^2+^ into the cytoplasm [90]. The increased Ca^2+^-level induced by RhoA/ROCK via PTEN/PLC activates pathological signaling by activation of CaMKII and CnA. While CaMKII inhibits hypertrophy-suppressing factors, Calcineurin promotes translocation of NFAT into the nucleus. NFAT induces genes that are associated with pathological hypertrophy (Reviews: [115, 172]) (Fig. 6).

In 2005 Pan et al. have shown that stretch-induced hypertrophy in NRVCMs is mediated by a signaling pathway involving RhoA/ROCK, PKCα and MAPKinases [122]. Furthermore, PKCα reduces cardiomyocyte contractility, as PKCα-knockout mice have hypercontractility, but PKCα-overexpressing mice show impaired contractility [18]. Even earlier experiments have shown that the MAPKinases ERK/MEK, p38, and JNK are activated by stretch-stimulation of NRVCMs [84] (Review: [86]). Furthermore, inhibition of ROCK with the specific inhibitor Y-27632 blocked the activation of various MAPKinases including ERK/MEK, p38 and JNK in vitro [30]. Consistently, RhoA-depletion in mice inhibited the TAC-induced activation of p38 [90]. The MAPK-signaling pathways activated by RhoA/ROCK via PKCα including MAPK/p38 and MAPK/JNK, can promote the translocation of GATA-4 and MEF2-A into the nucleus, respectively. There, GATA-4 and MEF2A act as cofactors for NFAT and promote the expression of pro-hypertrophic genes [115] (Fig. 6). In addition, the translocation of GATA-4 into the nucleus is associated with sarcomeric gene expression (e.g. *MYH6*, *MYH7* and *ACTC1*) which promotes actin polymerization and is also associated with cardiomyocyte hypertrophy [24]. In fact, experiments in cardiomyocytes have shown that GATA-4 is necessary for ET1 and PE-induced sarcomere reorganization [24]. Interestingly, the increase in DAG by RhoA/ROCK-induced PTEN/PLC-activation provides a possible pathway, by which RhoA can also act upstream from PKCα and function as its activator [90] (Fig. 6).

Furthermore, as mentioned above, RhoA strongly activates SRF-signaling. The activation of SRF includes its phosphorylation and translocation into the nucleus. One way of RhoA-induced SRF-activation is via the PKCα-mediated activation of the MAPKinases ERK1/2 (= MEK1/2) [128] (Review: [115]) (Fig. 6). Another way is RhoA/ROCK-mediated activation of MAPK/p38, which has also been shown to phosphorylate and translocate SRF into the nucleus [90] (Fig. 6). In addition to the promotion of SRF translocation into the nucleus, RhoA can increase SRF activity even more by stimulating co-activators of SRF. An important co-activator of SRF is the “myocardin-related transcription factor A” (MRTF-A, also named MKL1). In experiments in mice we have shown that TAC-induced RhoA activation is associated with activation of this co-activator MRTF-A [128] and MRTF-A has been proposed before to function as a link between ERK1/2 and SRF-activation [75, 110]. Furthermore, MRTF-A has a very high affinity for globular-actin (= G-actin) and binding to the monomer keeps it in the cytoplasm. Not G-actin bound MRTF-A in contrast translocates into the nucleus, where it binds and activates SRF [90]. As RhoA activation stimulates and stabilizes actin-polymerization, it reduces the amount of G-actin in the cytoplasm, thus increasing the translocation of MRTF-A into the nucleus and the activation of SRF. SRF binds to a specific gene sequence motif, called CArG-Box and is an established and very potent activator of the expression of fetal genes e.g. *NppA*, *NppB*, *ACTA1* and *MYH6* [128] (Fig. 6). In addition, SRF induces the expression of fibrosis-associated genes such as *collagene1a2*. This again fits well with the findings of RhoA-deficient mice, which show a reduction in TAC-induced fibrosis [90]. Furthermore, SRF activation starts a positive feedback loop, as one of the main target genes of this transcription factor is SRF itself [115]. In contrast, ANF and BNP, the protein products of *NppA* and *NppB* provide a negative feedback loop, as the activation of the natriuretic peptide receptor leads to activation of PKG and subsequently to the inhibition of RhoA and Calcineurin [115].

## The role of hypertension for RhoA-signaling in cardiac-hypertrophy

Hypertension is another key factor in the development of cardiac hypertrophy and the underlying mechanisms of high blood pressure and its links to hypertrophy and heart failure are still being extensively studied [20, 74, 115, 178]. Hypertension directly surge cardiac stress, leading to left ventricular hypertrophy in case of cardiac pressure-overload, or right ventricular hypertrophy in case of pulmonary hypertension [55]. Furthermore, hypertension correlates with the development of atherosclerosis and myocardial ischemia further accentuating the cardiac burden ensuing hypertrophic remodeling [74, 113]. On cellular level, hypertension promotes cardiomyocyte hypertrophy by generating biomechanical stress and through neuroendocrine hormonal receptor-stimulation (Fig. 4) [115, 178]. The control of blood pressure depends mainly on the contractility of vascular smooth muscle cells (VSMCs) and arterial wall thickening: increased vasoconstriction and reduced vasodilation lead to elevated peripheral vascular resistance that causes the arterial pressure-overload [12]. However, growth, contraction and relaxation of VSMCs are modulated by neural and hormonal signaling and the pathogenesis of hypertension involves multi-organ signaling networks, as for example the brain-renin-angiotensin system [64].

In vivo studies using experimental animals demonstrated that an increase in angiotensin II (AngII)-levels in the heart, circulating blood, blood vessels, and kidney as well as the activation of AngII receptor type 1 (AT1R) in the brain, lead to sympathetic activation and thus hypertension [64]. Experiments using RhoA/ROCK-inhibitors Y-27632 or fasudil suggested that this pressure response partly dependents on RhoA-signaling in the CNS [64, 71, 124, 138, 156]. Furthermore, RhoA/ROCK-activation in VSMCs has been shown to play an important role in hypertension [146, 156]. A number of studies analyzing data from in vitro and ex vivo experiments have shown that RhoA-activity is increased in VSMCs in different models of hypertension [91]. Mechanistically, RhoA/ROCK signaling has been shown to induce cardiac hypertrophy via AngII-receptor-mediated vasoconstriction, probably facilitated by RhoGEFs expressed in VSMCs (PDZ-RhoGEF, LARG and p115RhoGEF) [91, 156] (Figs. 3 and 4). In this context, RhoA has been suggested as a therapeutic target in hypertension [65, 111, 156] with positive effects also on cardiac hypertrophy [90, 143]. Importantly, studies with RhoA-inhibitor treatment of patients with pulmonary hypertension also demonstrated positive effects due to reduced blood pressure [119, 120, 156]. Taken together, the modulation of blood pressure and its interrelation with cardiac hypertrophy adds another level of complexity to the manifold RhoA-signaling in the cardiovascular system.

## RhoA-induced cardio-protective signaling

Although RhoA has been mainly described as inducer of detrimental effects, there is some evidence that RhoA can also induce cardio-protective signaling. RhoA activation in NRVCMs leads to activation of the “yes associated protein” (YAP). YAP is a transcriptional cofactor, which binds mainly to transcription factors of the TEAD-family [83]. Although YAP has been shown to get activated by signals exerted by tension of the acto-myosin cytoskeleton in epithelia cells [36], it has also been shown that YAP is not necessary for stretch-induced hypertrophic responses in NRVCMs [168].

On molecular level, RhoA-activation induces ROCK/LIMK which promotes polymerization of actin and stabilizes F-actin via inhibition of cofilin (Fig. 6). In line with this, experiments showed that RhoA-inhibition in cardiomyocytes by C3 leads to impaired cytoskeleton polymerization in fibroblasts and cardiomyocytes [127, 131]. One possible way, how RhoA is linked to nuclear localization of YAP and the activation TEAD, is via the factor angiomotin (AMOT) [38]. AMOT binds to YAP, retaining it in the cytoplasm, but can also bind F-actin instead [38]. So, the RhoA-induced increase in F-actin leads to release of YAP from AMOT and its translocation into the nucleus [38] (Fig. 6). Generally, TEAD target genes are involved in the heart in cardiomyocyte differentiation, cell growth, proliferation and anti-apoptotic signaling pathways. One of the genes regulated by YAP/TEAD is CCN1 (= Cyr61), a growth factor inducible early gene, which is associated with upregulation of proliferation and survival signaling [20]. Taken together these data suggest a cardio-protective role of YAP activation by RhoA in cardiomyocytes.

Another transcription factor, which is associated with CCN1-expression, is SRF. SRF-activation can induce CCN1, CCN2 (= CTGF) and “immediate early genes” such as c-fos, which are all associated with proliferation and cell survival signaling via integrins, FAK and AKT [20]. Upstream of YAP and SRF, the increased CCN1 expression is modulated by a variety of signaling pathways including RhoA/ROCK-mediated activation of PKC and the MAP-Kinases p38 and ERK [32, 70]. These pathways may also play a role in adaptive hypertrophy, as it has been shown that short-term activation of AKT induces compensatory hypertrophy in cardiomyocytes [90, 115]. In contrast, chronic activation of AKT leads to pathological hypertrophy and heart failure (Fig. 6). Similarly, SRF-activation is mainly associated with maladaptive hypertrophy and impairment of heart function [115] (as described above) (Fig. 6).

Another pathway that is modulated by RhoA and associated with cardio-protective responses involves the activation of PTEN and PLC leading to increased cytoplasmic Ca^2+^ level. The increase in cytoplasmic Ca^2+^ level promotes, not only pathological signaling such as CaMKII and CnA (as described above), but in contrast cardiomyocyte contractility [90] (Fig. 6). Furthermore, the RhoA/ROCK-induced activation of PI3K has been shown to promote cell survival signaling via AKT-activation [115] (Fig. 6).

## Links between RhoA and the immune system in cardiac hypertrophy

RhoA-depending signaling has also been shown to be essential for immune cell responses which may directly or indirectly impact cardiomyocytes and neighboring cells. A link between immune responses and cardiomyopathies, including cardiac hypertrophy, was discovered in 1990, when Levin et al. observed elevated cytokine levels in patients with heart failure [95]. Over the years, the involvement of the immune system in cardiac diseases has become an increasingly comprehensive field of research (Reviews: [42, 44, 69, 142].

Recent studies have shown that strict spatio-temporal regulation RhoA-activation is essential for directed migration of cells of the innate immune system, namely macrophages, dendritic cells, and granulocytes, as well as B- and T-cells of the adaptive immune system (Review: [19]). In this context RhoA-expression modulates extension and infiltration at the leading edge (pseudopod) [13], as well as detachment and retraction of the rear end (uropod) [13, 25]. In general, RhoA expression is induced in the rear end and inhibited in the front, but temporal regulation is crucial [13, 19, 25].

**Fig. 7:**
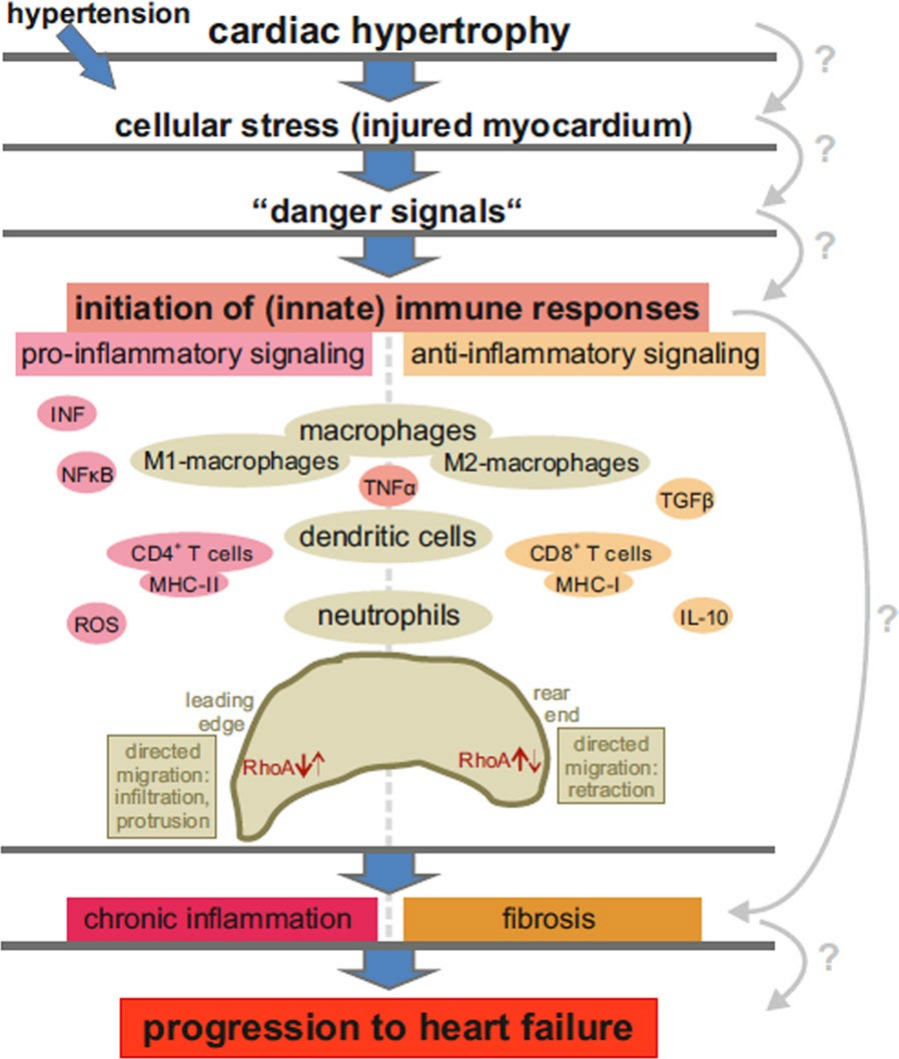
Links between RhoA-signaling, cardiac hypertrophy, and the immune system. Cardiac hypertrophy, in combination with other risks factors such as hypertension, leads to cellular stress and even injured myocardium. The damaged cells can release “danger signals”, which initiate an immune response. The immune response involves mainly cells of the innate immune system, namely macrophages, dendritic cells and neutrophils, and initiates the release of additional cytokines. The immune response includes pro- and anti-inflammatory signaling, but ultimately it leads to chronic inflammation, fibrosis, and progression to heart failure. For an effective response spatio-temporal regulated RhoA-expression in the immune cells is crucial. Details of the links between cardiac hypertrophy, the initiation of immune responses, and the subsequent deterioration of heart function are still unclear

There is some evidence that RhoA signaling also affects macrophage polarization, probably via its effector ROCK [98]. Principally two types of macrophages are defined: Firstly, classically activated macrophages (M1), which are mostly associated with pro-inflammatory signaling and secondly alternatively activated macrophages (M2), which are mostly associated with anti-inflammatory responses [114]. The majority of macrophages in damaged (cardiac) tissue originate from precursor cells circulating in the blood stream and present M1-characteristics [69]. However, also resident cardiac macrophages (rcMACs) have been described, which reside in healthy myocardium, get activated directly by signals of surrounding cells and present a predominantly M2-phenotype [69, 142]. A number of studies have demonstrated a correlation between the number of activated macrophages in the myocardium and the progression of cardiac hypertrophy to fibrosis and heart failure [37, 42, 51, 101, 123] (Fig. 7). However, the details of the links between hypertrophic cardiomyocytes, the initiation of the immune system and the subsequent progression of heart failure remain nebulous (Fig. 7).

Similar to cardiomyocytes, different GEFs, GAPs and GDIs have also been identified as regulators of RhoA-activity in immune cells [19]. Many of these modulators and signaling pathways match the ones described in cardiomyocytes, such as (e.g. LARG-RhoGEF, Lfc/GEF-H1 and PDZ-RhoGEF, p190RhoGAP and Vav1) [39, 155] and the initiation of RhoA-activation via membrane receptors coupling to Gα12/13 and RhoGEF-stimulation [41, 157]. The (up-)regulation of RhoA-activity by extracellular signals (e.g. via GPCRs) are particularly interesting, as it might present a way to link signals released by (stressed or damaged) cardiomyocytes to the activation of immune cells.

It is known that pattern recognition receptors (PRRs), which are commonly expressed by immune cells, can get activated by cytokines or other “danger” signals from stressed or damaged myocardium and thus induce an (innate) immune response [42, 97]. In addition, PRRs have also been found to be expressed by cardiomyocytes themselves [118, 179].

Furthermore, it has been shown that the natriuretic peptides ANP (*NppA*) and BNP (*NppB*), which as part of the “fetal gene program” are reactivated in the hypertrophic heart and associated via SRF with RhoA-activation [115, 128] can function as cytokines (Review: [31]). Macrophages express these natriuretic peptides themselves, but they also have membrane receptors for BNP and ANP [31]. Conversely, inflammatory cytokines, commonly released by (activated) immune cells (e.g. TNF and IL-1β) have been shown to induce upregulation of BNP in NRVCMs [121]. So, upregulation of BNP and ANP in hypertrophic cardiomyocytes might be directly linked to activation of immune cells and vice versa. Contrary, upregulation of ANP in NRVCMs has been demonstrated to reduce NFB-/TNF-induced immune responses [26, 42, 79, 109], highlighting the complex and often ambiguous role of RhoA-activation in the context of cardiac hypertrophy and immune responses.

## Perspectives

Although in vitro and in vivo data strongly suggests direct or indirect involvement of RhoA in cardiac pathophysiology, human and clinical studies are still lacking that support this notion. Also, no human mutations in the RhoA genes associated with cardiac mis-function are known so far. For Rac1 it has been shown that its activity is increased in failing myocardium of human patients suffering from dilated cardiomyopathy (DCM) or ischemic cardiomyopathy (ICM) [102]. This strengthens the hypothesis derived from the data of in vitro and in vivo models that RhoA is differentially regulated and probably up-regulated in patients with pathological hypertrophy. Nevertheless, it is misguiding to draw direct conclusions from the role of other SmG-proteins, even from the same family, as for example for Rac1 and RhoA converse effects have been shown in fibroblasts. While RhoA was shown to promote the formation of stress fibers and a fibroblastoid phenotype, Rac1 activation promoted spreading and an epithelioid phenotype, and even inhibited RhoA activation [141].

As it is known that PTMs of RhoA are essential for its function, the inhibition of different steps of these modifications has been analyzed with regard to effects on RhoA function in tumor cells and cardiomyocytes. GGTase1 inhibitors for example block the geranylgeranylation of RhoA and have been shown to inhibit RhoA function efficiently [2, 5, 150]. Although these inhibitors have been shown to reduce proliferation of endothelial and other cell types [78, 148] and diminish the growth of atherosclerotic plaques in vitro [5], they have also been shown to induce apoptosis [149]. However, as geranylation by GGTase1 is a common modification of a wide variety of proteins (e.g. other Rho/Rac/Cdc42-family members), for a practical therapeutic approach the specificity is insufficient and serious side effects are probable. Furthermore, GGTase1 and FTase can compensate for each other’s loss of activity if one is inhibited [147].

Statins are very commonly used to lower cholesterol-levels in the therapy of diabetes, but also inhibit the isoprenylation of SmGs [5]. Interestingly in vitro experiments with NRVCMs revealed an inhibitory impact of commonly used statins on AngII-induced Rac1 and RhoA activation [89]. Furthermore, the statin treatment reduced the hormone induced upregulation of the fetal gene *NppA* [89]. These findings are strengthened by findings on Rac1 activation in human failing heart. Treatment of patients suffering from ICM with the statins pravastatin and atorvastatin reduced Rac1-GTPase activity compared to control patients without statin-medication [102]. However, as mentioned above, to deduce the function of RhoA from data about Rac1 remains questionable and focused studies on RhoA activity are needed.

It is notable that, although there are no mutations of RhoA associated with cardiac diseases known yet, there are several activating and inhibiting mutations of RhoA described in different cancer types, as reviewed by Kim et al. [82]. For example, the RhoA-G17V mutation leading to increased activation of the RhoA-GEF Vav1 and a gain-of-function mutation in the RhoA-GEF Vav1 itself leading to increased RhoA activation, are found in T-cell lymphomas [27, 45]. Dasatinib is a multikinase inhibitor, which impairs Vav1-activation. It has already been used in the treatment for leukemia and has been shown to reduce T-cell activation in lymphoma cells [45, 77]. So, dasatinib might also have beneficial effects in cardiac hypertrophy, by reducing RhoA-activation in cardiomyocytes and/or restraining detrimental immune responses correlated with the progression to heart failure [94]. In cancer therapy a drug candidate named "Rhosin" has been designed, which effectively inhibits the Rho-subfamily of Rho GTPases, without affecting Rac1 or Cdc42 [153]. In in vitro experiments Rhosin-induced inhibition of RhoA lead to decrease proliferation of different cancer cells lines [153]. Rhosin is also tested in an drug-eluting stent as a potential method against arterial restenosis, by inhibiting neointima formation via blocking of RhoA-induced YAP activation and subsequent reduction of proliferation and survival signaling [68]. In respect to the functions of RhoA in cardiomyocytes, the effects of this inhibitor on heart function in cardiac hypertrophy or other heart diseases could be subject of future research.

In the last years the structure of RhoA and the biochemical mechanisms of its function as a molecular activatior have been studied in detail with promising results. Furthermore, the RhoA effector ROCK has been studied extensively. Nevertheless, many aspects of the regulation of RhoA, of downstream pathways and consequently cellular responses, and effects on heart function still remain unclear. It has been shown that RhoA plays some role in cardio-protective signaling, especially adaptive hypertrophy, but the detrimental effects of RhoA activation in cardiomyocytes predominate and it is associated with pathological hypertrophy and heart failure. Thus, more research focusing on the physiological function of RhoA as well as its distinct role in adaptive and maladaptive cardiac hypertrophy is needed. Furthermore, the study of the expression level and the activation of RhoA in human patients suffering from genetic cardiomyopathy, DCM or ICM would be a promising starting point for further research. For further in vitro and in vivo-experiments special focus should be put on differentiating between different cell types, to characterizes e.g. cardiomyocyte- or fibroblast-specific effects of RhoA modulation. These future studies should result into new therapies for pathological cardiac hypertrophy, by inhibition of detrimental pathways and/or enhancement beneficial signaling pathways downstream of RhoA, which are associated with adaptive hypertrophy or other cardio-protective effects.

## Conclusion

In conclusion, our understanding based on the current available data on RhoA suggests it being both benign and damaging to the cardiac function based on its expression levels and the state it exists. Thus, the fine tuning of the RhoA activation is essential for cardiac homeostasis. Summary of the existing data in this review presents promising starting points for further research, with regard to the molecular mechanisms of RhoA-signaling in cardiac pathophysiology and the corresponding cellular responses and clinical relevance. One of the immediate directions of further research could be assessing the expression and activation levels of RhoA in human patients suffering from genetic cardiomyopathy, DCM or ICM. Furthermore, RhoA-signaling provide an interesting target for new therapies by inhibition of detrimental pathways and/or enhancement of beneficial signaling pathways downstream of RhoA, which are associated with adaptive hypertrophy or other cardio-protective effects.

## Data Availability

Not applicable.
